# Supplementary figures and images for: Increased chemical acetylation of peptides and proteins in rats after daily ingestion of diacetyl analyzed by Nano-LC-MS/MS
Source: PeerJ. 2018 Apr 25;6:e4688. doi: 10.7717/peerj.4688 (PMC5923218; doi:10.7717/peerj.4688)

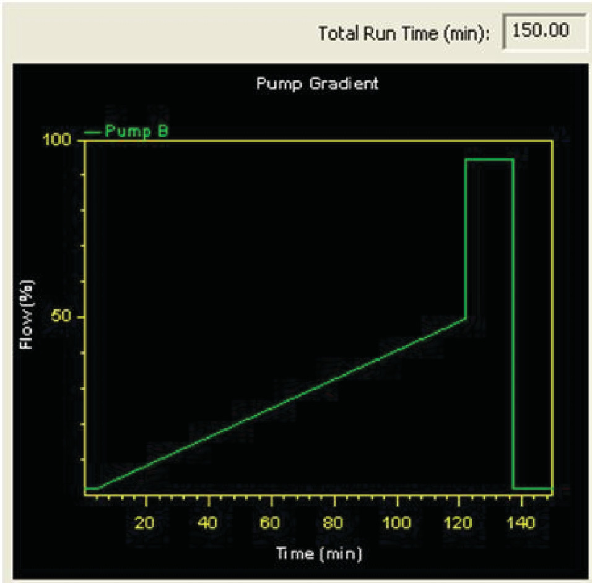

Supplement: Supplemental Information 1 — NanoHPLC gradient elution, gradient mobile phase consisting of (A) formic acid/ACN/ H2O (1:20:980) and (B) formic acid/ACN/ H2O (1:950:50). [file peerj-06-4688-s001.png]
