# Supplementary material for: Increased chemical acetylation of peptides and proteins in rats after daily ingestion of diacetyl analyzed by Nano-LC-MS/MS
Source: PeerJ. 2018 Apr 25;6:e4688. doi: 10.7717/peerj.4688 (PMC5923218; doi:10.7717/peerj.4688)
Supplement: Supplemental Information 2 — Motif analysis of Lung from control group. Central residue H acetylated. [file peerj-06-4688-s002.docx]

|  |
| --- |

Parameters for this run:

Job Name = **Lung Control Acetylati H**

fgtype = 'ms'

fgextenddb = 'ipi.RAT.fasta'

fgcentralres = 'H#'

width = '15'

occurrences = '7'

significance = '0.000001'

bgdb = 'ipi.RAT.fasta'

bgtype = 'fasta'

bgcentralres = 'H'

Job started Wed Feb 3 13:52:19 2016

Results for (**Central Foreground Residue: H# ; Background Residue: H**)

Number of Peptides in Original Dataset: 5114

Number of Peptides in Orignial Dataset that are Unique: 860

Number of Peptides found in Database (ipi.RAT.fasta): 842

Number of Peptides NOT found in Database (ipi.RAT.fasta): 18

Number of central residues (residue = 'H#') mapped to the database : 1281

Number of peptides without unique database mappings: 8

Number of peptides too close to protein termini: 25

Final Unique Target Peptides: 908

It took 112 seconds to preprocess foreground dataset

The input file has been converted to a pre-aligned file that may be used for subsequent runs of motif-x.

[Right-click here](http://motif-x.med.harvard.edu/cgi-bin/viewres.pl/motif-x_20160203-760-07599868_Hx_vs_H-2031_fg.txt?text=n) to save it as a 'pre-aligned' dataset for possibly faster analysis in the future.

It took 3 seconds to preprocess background dataset

**Motifs Found**

| \| **#** \| **Motif Logo** \| \| --- \| --- \| \| 1. \| [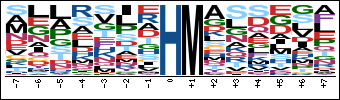](http://motif-x.med.harvard.edu/cgi-bin/jobres.pl?jobid=20160203-760-07599868#.......hM......) \| | \| **#** \| **Motif** \| **Motif Score** \| **Foreground Matches** \| **Foreground Size** \| **Background Matches** \| **Background Size** \| **Fold Increase** \| \| --- \| --- \| --- \| --- \| --- \| --- \| --- \| --- \| \| 1. \| **[.......hM......](http://motif-x.med.harvard.edu/cgi-bin/jobres.pl?jobid=20160203-760-07599868#.......hM......)** \| 6.17 \| 43 \| 908 \| 6943 \| 338541 \| 2.31 \|   Motif search time: 6 seconds |
| --- | --- | --- | --- | --- | --- | --- | --- | --- | --- | --- | --- | --- | --- | --- | --- | --- | --- | --- | --- | --- | --- |

**Raw sequence data** (motifs followed by their matching peptides):

.......hM......

MGPLGLDHMASSIER

NGARVPSHMSSSHSF

MDPRVLHHMGGMAGL

SHLRHIEHMESDELG

EMPRSIVHMHSDMPG

KTARKPEHMAVEIPP

MKAEETCHMKSSDKE

HCTRNYIHMHLFMSF

LILSHIRHMSNKGME

SGHSGPGHMMNRGGM

MLPQQNDHMYRQTEN

GENQTIRHMAKYFST

KNGLSPLHMATQGDH

VLSANADHMAQIEGL

QEDLEITHMYSAEGE

DQPDNIAHMVAKGAA

CQLAQEEHMLKSESC

FISEVTVHMNSEELF

NLLEKLGHMDLSSRL

SERRVVAHMPGDIII

SAVMMLRHMGLFDHA

ICPITDDHMSNQNVN

ANEMMMYHMKVSDDE

AGAAISSHMDVDKVA

EELDESRHMTQEVVT

VLFNDGTHMALSANR

NQTFSDCHMELNENT

TILLSTHHMDEADLL

MADTFLEHMCRLDID

AYLPQYTHMQTATVP

TSASVSTHMTAGAMA

DLSSHNNHMAKVLTP

DDTKGPGHMFGHLDF

SNAVGEIHMKTMPAA

CAYGFRAHMMNLGSY

EVTSKRAHMLIDMHF

VRGETALHMAARSGQ

ANNHPSMHMGQPSCP

LYLASLRHMGDGHQQ

AEGKLDHHMNSVLIQ

NGGDLMFHMQRQRKL

GLSHFCEHMLFLGTK

SLQNTKSHMCGGVLV

Please cite:

Chou MF & Schwartz D (2011).

Biological sequence motif discovery using motif-x.

Curr Protoc Bioinformatics. Chapter 13:Unit 13.15-24. doi:10.1002/0471250953.bi1315s35.

Schwartz D & Gygi SP (2005).

An iterative statistical approach to the identification of protein phosphorylation motifs from large-scale data sets.

Nature Biotechnology 23(11):1391-1398.

Parte superior do formulário

Parte inferior do formulário
