# Supplementary material for: Increased chemical acetylation of peptides and proteins in rats after daily ingestion of diacetyl analyzed by Nano-LC-MS/MS
Source: PeerJ. 2018 Apr 25;6:e4688. doi: 10.7717/peerj.4688 (PMC5923218; doi:10.7717/peerj.4688)
Supplement: Supplemental Information 3 — Motif analysis of Lung from group treated with 540 mg/Kg/day of Diacetyl. Central residue H acetylated. [file peerj-06-4688-s003.docx]

|  |
| --- |

Parameters for this run:

Job Name = **Lung Treated Acetil K**

fgtype = 'ms'

fgextenddb = 'ipi.RAT.fasta'

fgcentralres = 'H#'

width = '15'

occurrences = '7'

significance = '0.000001'

bgdb = 'ipi.RAT.fasta'

bgtype = 'fasta'

bgcentralres = 'H'

Job started Wed Feb 3 14:19:32 2016

Results for (**Central Foreground Residue: H# ; Background Residue: H**)

Number of Peptides in Original Dataset: 6181

Number of Peptides in Orignial Dataset that are Unique: 1313

Number of Peptides found in Database (ipi.RAT.fasta): 1276

Number of Peptides NOT found in Database (ipi.RAT.fasta): 37

Number of central residues (residue = 'H#') mapped to the database : 1712

Number of peptides without unique database mappings: 15

Number of peptides too close to protein termini: 46

Final Unique Target Peptides: 1206

It took 37 seconds to preprocess foreground dataset

The input file has been converted to a pre-aligned file that may be used for subsequent runs of motif-x.

[Right-click here](http://motif-x.med.harvard.edu/cgi-bin/viewres.pl/motif-x_20160203-13130-61802867_Hx_vs_H-32336_fg.txt?text=n) to save it as a 'pre-aligned' dataset for possibly faster analysis in the future.

It took 2 seconds to preprocess background dataset

**Motifs Found**

| \| **#** \| **Motif Logo** \| \| --- \| --- \| \| 1. \| [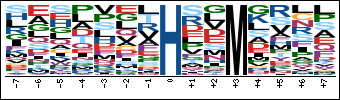](http://motif-x.med.harvard.edu/cgi-bin/jobres.pl?jobid=20160203-13130-61802867#.......h..M....) \| \| 2. \| [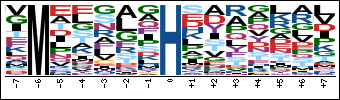](http://motif-x.med.harvard.edu/cgi-bin/jobres.pl?jobid=20160203-13130-61802867#.M.....h.......) \| \| 3. \| [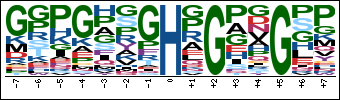](http://motif-x.med.harvard.edu/cgi-bin/jobres.pl?jobid=20160203-13130-61802867#.......h.G..G..) \| | \| **#** \| **Motif** \| **Motif Score** \| **Foreground Matches** \| **Foreground Size** \| **Background Matches** \| **Background Size** \| **Fold Increase** \| \| --- \| --- \| --- \| --- \| --- \| --- \| --- \| --- \| \| 1. \| [**.......h..M....**](http://motif-x.med.harvard.edu/cgi-bin/jobres.pl?jobid=20160203-13130-61802867#.......h..M....) \| 7.50 \| 56 \| 1206 \| 6946 \| 338541 \| 2.26 \| \| 2. \| [**.M.....h.......**](http://motif-x.med.harvard.edu/cgi-bin/jobres.pl?jobid=20160203-13130-61802867#.M.....h.......) \| 7.08 \| 52 \| 1150 \| 6596 \| 331595 \| 2.27 \| \| 3. \| [**.......h.G..G..**](http://motif-x.med.harvard.edu/cgi-bin/jobres.pl?jobid=20160203-13130-61802867#.......h.G..G..) \| 13.32 \| 28 \| 1098 \| 1718 \| 324999 \| 4.82 \|   Motif search time: 6 seconds |
| --- | --- | --- | --- | --- | --- | --- | --- | --- | --- | --- | --- | --- | --- | --- | --- | --- | --- | --- | --- | --- | --- | --- | --- | --- | --- | --- | --- | --- | --- | --- | --- | --- | --- | --- | --- | --- | --- | --- | --- | --- | --- |

**Raw sequence data** (motifs followed by their matching peptides):

.......h..M....

MISDEERHRQMIRNY

QELTHQEHRVMLVNS

AQEPSAAHRSMGYCP

SNRPVCRHFMMKGNC

SGCISQTHDVMEMIF

QQHKQELHKLMARCF

CFIISLPHFLMGRYE

YETTVVRHGLMLVGP

HGWMFAEHGKMSRST

KEWRHAAHHVMGPPL

DACLSIVHSLMCHRQ

LAPAEGTHPGMAAPQ

RASSWWTHVEMGPPD

GQLYDMFHSVMKYLP

IEETMSVHDIMKAFQ

KILLNIEHRIMLRMA

HFWGFQTHDTMPDIV

GHHTPFMHLGMAHNY

MGCPPSLHQLMLHCW

ESQDSGAHRDMTRVP

HIGDLLQHRHMLVRM

GMLSKLCHNEMCRCA

LEVDQLRHSLMKAED

FEVLEPLHAMMERGP

SEEELQKHEGMSERE

RLQAMMTHLHMRPSE

KAALRKAHTSMVRNF

DLYPNQSHPSMMYRS

FYRQSEGHSMMDTLA

SSAPGECHQVMKNLQ

SASQVSVHVGMDDRR

RQYELVVHEDMDVAK

QGIMVHKHEGMKVFV

GDGNNILHSIMMSAA

IPVLPTVHYNMGGIP

LEHHHFNHAVMILQS

IVDPVEPHGEMKFQW

LMHKPLRHPGMPKGL

PLKQFCDHLDMGCSQ

VFITDDFHDMMPKYL

MDGLHGGHISMSNLT

RTSQLRRHMQMSSQE

HLAAQEGHVDMVSLL

SDMNRHLHEYMEMCS

HTSSVFFHSPMHEYA

TGKAMVPHSSMFIFS

TKENDGFHRFMNSAK

INSPVTDHVEMMLTE

CPKIGCSHTDMRMSD

LPGGIDTHTHMQFPF

FSPAAGVHSPMGSSG

RSQFPVLHMEMIVHL

PNHVKGLHLNMAFIS

EKQDPLSHFNMDTLL

VAEERNIHYNMSSFN

VTFRLDTHPAMVTVL

.M.....h.......

IMESSGPHSDRNMAV

IMTMLADHAARQLLD

GMAGGAVHIDYNCVP

VMICSGHHVYPHLPK

KMICCAPHDSNAERH

GMESCGIHETTFNSI

MMEPSEYHEYQARGK

KMFNALIHRYRPDLV

TMTLYLRHYWRDERL

IMRLSSLHKDRPIEP

IMAVLFFHTMRYKAL

TMQQQAQHAIATEDQ

FMHLGMAHNYRNSSQ

GMPFRQAHEASGKAV

YMEGNLGHPVFQTQF

VMLLSTIHHDQPMKP

QMNARFKHENYGLMP

VMMQGRFHMYEGYSL

VMNLMDAHKVFQKEL

VMKEMGGHHIVALCV

FMGGWFAHPIFKNGD

VMELLLKHGASIQAV

MMIKNVLHSARLLGD

DMTSHSQHSTATRQP

SMDENLMHISYEAGI

KMQGRRYHGVTGLVV

PMPSVAPHSALGATG

VMEAAAKHLTPVTLE

QMDEGLSHITPRDDI

EMDINECHSDPCQND

RMEEEQHHWDDRRRM

LMTDEIFHDVAYKAK

FMQRCASHMKAVIQG

GMVGGGGHRPHEGPG

CMIPRDTHDGAFYRA

HMLRVFMHDNSQGRI

GMPKGLAHQLASPGL

GMVKILGHKSTDRIL

EMLMWSDHPEYPFHD

AMVYIRGHAEDYNRW

QMLNVTPHHHQHSHI

LMSTLLIHHPQYAWL

TMDEFRQHLQATGVS

HMNSSGSHLGQMTMT

IMSTASQHPAVVGDS

EMTENGSHQLIVKAR

LMEYAKQHGIPIPVT

NMAVFHFHYAGDRTM

TMRTFALHKSGSSEK

AMATRTRHEYLKDLA

RMAIVADHLGLSWTE

SMPAFKGHSFLAFPT

.......h.G..G..

GSPGAAGHQGAVGSP

GEPGPQGHAGAQGPP

GHPGAAGHPGEQGQP

GKPGASGHNGERGPP

GRKGHKGHPGAAGHP

GKDGSSGHPGPIGPP

GPPGSSGHPGSPGSP

GFPGMKGHRGFDGRN

DGHGGGGHRGHDGGH

GGGGHRPHEGPGGSM

GPKGSMGHPGTPGGV

GGPMPGPHGGPGGPV

MGSGHRSHEGPGGSM

RSHEGPGHGGPHGHR

GLAGYNGHKGITGPL

KPPPPPPHRGPVGNW

PPHEHRGHDGHGGGG

CWGKIGGHGGEYGEE

RGPPPHEHRGHDGHG

MDQYGGRHGGGSGGP

KTKAFVTHGGANGLY

DTVKQYFHAGGNGLK

ATIKQYFHAGGNGLK

MRECISIHVGQAGVQ

TSINVVRHLGIVGEC

LGNLRPAHLGPCGDG

ERFSLYQHQGGLGSQ

KARAFITHSGSHGIY

Please cite:

Chou MF & Schwartz D (2011).

Biological sequence motif discovery using motif-x.

Curr Protoc Bioinformatics. Chapter 13:Unit 13.15-24. doi:10.1002/0471250953.bi1315s35.

Schwartz D & Gygi SP (2005).

An iterative statistical approach to the identification of protein phosphorylation motifs from large-scale data sets.

Nature Biotechnology 23(11):1391-1398.

Parte superior do formulário

Parte inferior do formulário
