# Supplementary material for: Increased chemical acetylation of peptides and proteins in rats after daily ingestion of diacetyl analyzed by Nano-LC-MS/MS
Source: PeerJ. 2018 Apr 25;6:e4688. doi: 10.7717/peerj.4688 (PMC5923218; doi:10.7717/peerj.4688)
Supplement: Supplemental Information 4 — Motif analysis of Lung from group treated with 540 mg/Kg/day of Diacetyl. Central residue K acetylated. [file peerj-06-4688-s004.docx]

|  |
| --- |

Parameters for this run:

Job Name = **Lung Treated Acetil K**

fgtype = 'ms'

fgextenddb = 'ipi.RAT.fasta'

fgcentralres = 'K#'

width = '15'

occurrences = '7'

significance = '0.000001'

bgdb = 'ipi.RAT.fasta'

bgtype = 'fasta'

bgcentralres = 'K'

Job started Wed Feb 3 14:15:08 2016

Results for (**Central Foreground Residue: K# ; Background Residue: K**)

Number of Peptides in Original Dataset: 6181

Number of Peptides in Orignial Dataset that are Unique: 1805

Number of Peptides found in Database (ipi.RAT.fasta): 1768

Number of Peptides NOT found in Database (ipi.RAT.fasta): 37

Number of central residues (residue = 'K#') mapped to the database : 2133

Number of peptides without unique database mappings: 54

Number of peptides too close to protein termini: 63

Final Unique Target Peptides: 1764

It took 51 seconds to preprocess foreground dataset

The input file has been converted to a pre-aligned file that may be used for subsequent runs of motif-x.

[Right-click here](http://motif-x.med.harvard.edu/cgi-bin/viewres.pl/motif-x_20160203-11414-12570355_Kx_vs_K-18844_fg.txt?text=n) to save it as a 'pre-aligned' dataset for possibly faster analysis in the future.

It took 4 seconds to preprocess background dataset

**Motifs Found**

| \| **#** \| **Motif Logo** \| \| --- \| --- \| \| 1. \| [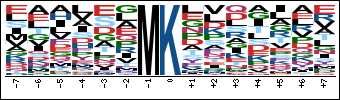](http://motif-x.med.harvard.edu/cgi-bin/jobres.pl?jobid=20160203-11414-12570355#......Mk.......) \| \| 2. \| [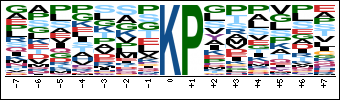](http://motif-x.med.harvard.edu/cgi-bin/jobres.pl?jobid=20160203-11414-12570355#.......kP......) \| \| 3. \| [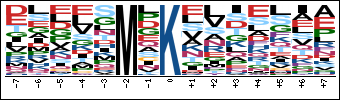](http://motif-x.med.harvard.edu/cgi-bin/jobres.pl?jobid=20160203-11414-12570355#.....M.k.......) \| \| 4. \| [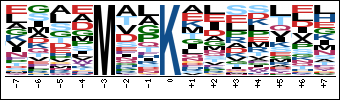](http://motif-x.med.harvard.edu/cgi-bin/jobres.pl?jobid=20160203-11414-12570355#....M..k.......) \| | \| **#** \| **Motif** \| **Motif Score** \| **Foreground Matches** \| **Foreground Size** \| **Background Matches** \| **Background Size** \| **Fold Increase** \| \| --- \| --- \| --- \| --- \| --- \| --- \| --- \| --- \| \| 1. \| [**......Mk.......**](http://motif-x.med.harvard.edu/cgi-bin/jobres.pl?jobid=20160203-11414-12570355#......Mk.......) \| 16.00 \| 112 \| 1764 \| 16508 \| 705467 \| 2.71 \| \| 2. \| [**.......kP......**](http://motif-x.med.harvard.edu/cgi-bin/jobres.pl?jobid=20160203-11414-12570355#.......kP......) \| 13.21 \| 169 \| 1652 \| 38368 \| 688959 \| 1.84 \| \| 3. \| [**.....M.k.......**](http://motif-x.med.harvard.edu/cgi-bin/jobres.pl?jobid=20160203-11414-12570355#.....M.k.......) \| 10.82 \| 76 \| 1483 \| 14053 \| 650591 \| 2.37 \| \| 4. \| [**....M..k.......**](http://motif-x.med.harvard.edu/cgi-bin/jobres.pl?jobid=20160203-11414-12570355#....M..k.......) \| 7.06 \| 60 \| 1407 \| 12748 \| 636538 \| 2.13 \|   Motif search time: 15 seconds |
| --- | --- | --- | --- | --- | --- | --- | --- | --- | --- | --- | --- | --- | --- | --- | --- | --- | --- | --- | --- | --- | --- | --- | --- | --- | --- | --- | --- | --- | --- | --- | --- | --- | --- | --- | --- | --- | --- | --- | --- | --- | --- | --- | --- | --- | --- | --- | --- | --- | --- | --- | --- |

**Raw sequence data** (motifs followed by their matching peptides):

......Mk.......

MVEIDGMKLVQTRAI

EDMLSQMKGVQQIDI

MVEIDGMKLAQTRAI

IQHLAGMKDSKTIVA

MVVLDPMKPGGPFEV

GRHFEQMKDDAIVCN

IMHYGNMKFKQKQRE

RGIASGMKYLSDMGY

RILRSPMKFFDTTPT

EALANNMKLKQDIAR

EPHVSGMKRSRSGEG

AQAHGRMKPLSASQV

MPGTTQMKSTDILYR

MSVHDIMKAFQSGRD

GTHYGLMKYIGEVVR

SACFSTMKETDLEAV

GECHQVMKNLQAHYE

EAAMEAMKGRLANQY

VDFNVPMKNNQITNN

GRIKGGMKGFQSFMV

IHHDQPMKPLDRAVF

MAILQIMKELNKRGR

VAHVQQMKTVKQSVL

EKTISGMKNIIAEME

MYQSSEMKGWPSGNL

DNGPEGMKGKPGARG

SSVISIMKPVRKRKT

MSNMTLMKETITTVE

VEHQVLMKTVCGTPG

IPGFPGMKGHRGFDG

NSASLQMKSPAITAT

DDMASAMKAVTELNE

HAFLILMKYPKVQAR

RVLVTVMKYFQWAHA

SAYMDPMKQNGGPLT

LNEASVMKGFTCHHV

LEIIEGMKFDRGYIS

VEPHGEMKFQWDLNA

SQGISKMKPPSQRCV

IGAFVLMKMKETAEN

AAADETMKLQSEEVE

SAHLERMKNNMEQTI

EDIDTAMKLGAGYPM

LEAETLMKNDEICGT

DMFHSVMKYLPGPQQ

TTACSLMKIANDIRF

LAAIEIMKLKHILIL

EVVREFMKDTDSINL

NTVVLMMKYYSAPWT

KIASAIMKAADEVAE

DQYVERMKEKQDKIY

AMTDHEMKGQTAILV

EIMSLQMKHTDLDHE

AEHPGFMKTGECLRC

KLIVALMKPSRLYDA

VSLPSPMKRPRLARA

ETLYYAMKGAGTDDH

KQVAGTMKLELAQYR

NVGIYKMKVPSTIAC

QNIIMAMKDRMKIRE

AGLCDAMKPIDGRKL

RLARDVMKEMGGHHI

EIDLDSMKNQNINLE

VIILGPMKDRINDDL

WKQFAQMKPDAPFLF

SLSVTPMKSPQAIQK

PGKVNGMKTSRASDN

AKQYGHMKIVALMET

VHKHEGMKVFVPTGF

LIGDVEMKRVLSCPR

LKDVAEMKEDLMRMT

QVTYAYMKNMWKSAR

SSYLEYMKVVDGLEK

QRCASHMKAVIQGFS

MLQKGFMKEELSVKR

LTINIRMKEDLIKEL

VCRHFMMKGNCRYEN

ITFTAMMKSAKLPQT

LLSANEMKLKRPARE

LGESLAMKEKTISGM

CAISILMKDLADELA

NPPKEAMKDDITGEP

FDAWNIMKSVPEGPV

TLYKDAMKNLLPTPA

GYDPRTMKHTTDLDA

PADPNRMKLLEALLG

SRQALEMKMESPTGA

KLLDSLMKTNFTGVS

HGFLLLMKHPDVEAK

DYDGPLMKTEVPGPR

RNRSPCMKDRSQMPY

REKPEDMKENGSPYN

TAELMEMKAHIQKVE

ILLAQQMKNLEVFMH

IFYVPEMKPSMFDVS

KTWRDVMKMVVQNKH

GEKSAAMKKQKVARR

PTDDIHMKQIDLENV

LLMTEVMKLQKKVQD

ENFEPFMKAMGLPED

ERMLERMKKSNELLE

KVFYYKMKGDYHRYL

ILRGAQMKASLAALP

QGQETIMKKVTSVKG

EELEERMKCPSILSQ

QGYQVTMKTAKVAAS

SQAVKAMKEENVKTV

VKNWPWMKLYFKIKP

AEVNRMMKLIINSLY

YMEMCSMKRGLDVQM

TTVEKEMKSLARKAM

SITFFRMKVLELQSP

.......kP......

GGPGNDGKPGPPGSQ

PAAPPIPKPPPPPPH

MAPAVPTKPLRMVHQ

PMSVDVPKPLPPVEL

RVETGVLKPGMVVTF

PQPPLSTKPSTAEPR

TPPPVSPKPPPPPTA

MTLTLKPKPSMGLSQ

RLVPGWTKPITIGRH

GPEGMKGKPGARGLP

GFPGARGKPGPMGKA

NTVEMMHKPRCGVPD

GTQGSASKPKPEDPR

LVIEDAPKPCVPVGM

SALNHSSKPLPMAPE

PSAYGSVKPYTNFDA

GADILMVKPGLPYLD

PVPSVSPKPETPVKC

ENGIKMCKPCTDICP

AASKAAAKPKTMPKE

ESLKPATKPLSSKSV

SEPKPSPKPLNLVSS

HMRPSEPKPSPKPLN

NKPGLKYKPVTNQVE

VKPPVSPKPILAQPV

NGRIVATKPLYVALA

GDHPWIPKPVQIPAT

INPQDYTKPIQEVLQ

YLSEFSLKPGTMLPP

SEGKLMCKPIFSKIV

FDQIVSSKPETFSFV

TAQSVSLKPGPVPEP

RPQFCVCKPGTKGKA

RWSFSMAKPATSSSS

EALKTSGKPIAATMC

GEEGPKGKPGKAGAS

PPPPTAPKPAKALAG

KVVEKKTKPYIQVDI

NLDSLVTKPAPPAQS

CGNAMIFKPSPFTPV

NKPGLKYKPVCNQVE

GMYVANTKPGGFTME

AMTSLKGKPSISGGV

IEMPMAFKPGEEFYP

MAGFLFTKPKVISLL

HFELNVFKPMMIKNV

AELKKQSKPVTTPEE

GPATAKVKPTPQLLP

LGSPTSPKPGANAQF

MEPGAGSKPRRPLLL

TKTPKFFKPAMPFDL

RARIDEFKPYIPLIQ

EPRSSSVKPSPISAP

AQEGGMVKPPGLWDL

PYNPSMSKPDAWGVT

LDMGCSQKPKAEQSE

ESQLSASKPTGGLPE

LSLVLMHKPLRHPGM

LMVTEALKPYSKGGP

GIKGESGKPGASGHN

IRDSGGPKPVMVYIH

AASHPAGKPQPGPTT

QLPPVPPKPRPLLSY

IAIADAVKPEAALAS

EMAAKGIKPITLELG

PNQTAVIKPFPKSTE

SKWESKSKPTTLKPI

HSVLGDFKPYRPGQA

HALPGTRKPHTVVVQ

RLQKAGPKPSSLSMA

MPDLRRKKPLPLVSD

CDLGAVTKPWEISRQ

GGELGSKKPVHPNDH

NQELKPTKPMRFLGD

VKPVKASKPKKAKPV

SFVMSYWKPEMMIAA

ERQKRILKPAMALQE

AQMTLTLKPKPSMGL

MNYVMLGKPMNNGLM

APDLLPQKPLPASAF

PTIISNVKPSMTCYK

ITLHSAQKPLRKVCI

ANQWQVSKPKDNPLN

TATPPGYKPGSPPSF

QSRAPEGKPIPTQRE

IVDMVPGKPMCVESF

ADSRESLKPATKPLS

TSGPTSEKPARSLPW

PGSQRCTKPSCVPPC

REMYSASKPLKGARI

DDHFLFDKPVSPLLT

GPKGGLTKPPAATRS

RTEGHEEKPLPPAQS

QGSASKPKPEDPRGV

VVEESDPKPAFSKMN

PLIKNLPKPIESLMT

NFGKLIIKPASEGTT

SKAYPSLKPLASWIM

QVPGAVAKPYHRQGA

ELMTFGGKPYDGIPT

PAPETAKKPQDAKSS

IRSFDVNKPGCEVDD

GKSYPSLKPLGSYVN

RPIPTTQKPTPLTSH

MSAKNAEKPNMQRNN

KGDGARSKPKVVTTT

PMEEDPEKPAESQPP

SGLDMHTKPWIRARA

GPPSSEDKPLCYKSV

QPAYRILKPWWDVFT

LLPPCMLKPPKRTHT

KDETVWEKPLRFHPE

KDLIYKAKPKDPFLK

KKGDGKSKPSAASPK

AHACVTGKPISQGGI

ECGIRCKKPSMLKKH

LCPDGTKKPVTEFAT

CLVEMSEKPYILEAA

FSLQKGWKPMVIVNR

VGSHSSTKPCYHQAK

GGCGYVLKPPVLWDK

KSGRFLSKPIFAKMA

ATATILDKPEDRVSV

EKRIKVEKPVVEMDG

LMVSRMAKPEEVLVV

LQFQQSPKPARAATT

ILTTSLSKPFMRLEK

RSHSWVKKPGSVCSL

FSLQIAWKPVVVING

AAHAPLEKPASTAIL

AGAHGDSKPVFVKVP

EKVMGGGKPWHKTCF

PTKRPPTKPSVSSLS

SLLTTHQKPSIFVGP

VVKARQPKPFFAAGN

KEVKACCKPGSHSQQ

SKKKEKSKPYPGAEL

SSDLWGEKPNTSYII

LQWLKNGKPVSTAWG

ESIMIGVKPCIDKNV

HLTLLGLKPDTTYDI

TPASHPKKPDCPTSS

VQQRAVCKPVGDICE

APSVEKEKPTREDSE

KVYIILAKPERNVRS

DAHKKEHKPKKPGKY

GSARKPVKPPVSPKP

STQHSDPKPHSNRPG

LGRFEKKKPDPLEIG

YPIAIFIKPKSLEPL

DDRSKIAKPSETLIT

EEIWVLRKPFAGGDR

KSKPTTLKPIILDEI

HTGEKPYKPYECVEC

QAIAERIKPVLMMNK

WEVITRRKPFDEIGG

EDSEGQEKPRVRSRS

LETLDNGKPYVISYL

GPLDTRLKPRNTTWL

GINCHQGKPLSKEFE

LIEQLQNKPSDLGTK

QTDVPEEKPFQTDLP

IRLLRLSKPDLPFLI

RVTEEPDKPEKEKEA

FLGVPFAKPPLGSLR

QRVHTGEKPYECVEC

QRIHTGEKPFRCSEC

QRIHTGEKPYQCIEC

RISHTGEKPYKCNEC

.....M.k.......

ELRLSMQKSMQSHAA

IADFGMCKENIFGEN

SLEKSMDKMMTEWEA

QEAVSMAKKNSEQRA

FRVVSMDKNLHPLNE

DEDIMMQKAVKKFAQ

TVDETMSKVEIWLIR

PPVLGMPKRRPQSQE

LKDCSMIKLVDIQIM

DYERSMNKINFDQIV

TLPKGMPKDVTELYL

DLLVPMPKASDLTVG

KMNGSMDKKSSTVSE

NSGLVMGKTRKNHIP

MRPSSMIKVIGQSAV

TAALPMLKQSNGSIA

DNGSGMCKAGFAGDD

DIDEKMGKKLKWFGN

EMVESMKKVAGMDVE

DENSKMEKSVIQGLF

RSEVDMLKIRSEFKR

EPLMEMNKRLTEEQA

VSSVTMSKNMLETSP

TLDLSMNKQRPRDSC

IPGMWMGKSYPSLKP

TGLVVMDKNNDRETD

INDLRMPKFSISTDY

DTEIIMTKKLQNQVN

NGPEIMSKLAGESES

EDKETMEKAVEEKIE

KECGMMDKNLDDEEF

VLGASMPKLYQHAQQ

EGCIDMCKSFHTSTI

DHLTLMQKVEVFEHA

MNNGLMEKMVEESDE

ILISTMNKLATAMQE

PQELNMGKISAEIMW

LRHPGMPKGLAHQLA

GPPGLMGKEGIIGPP

EMSQLMLKEPTHCEA

GIADRMQKEITALAP

QLQKGMVKEAIDSYI

MEEVHMIKNKPASPH

DTGIGMTKEELVSNL

VFKPMMIKNVLHSAR

PSQVNMLKTTKFNSE

PDIVTMAKGIGNGFP

VSGLEMRKWVDELFV

EVSWHMGKSLNFTVK

NGFSAMAKTVGLPTA

HDLEKMLKESSRNPE

TLLECMHKLGNSCEF

HCTCPMCKLNILKAL

YIVHVMSKSAAKVIA

EEVFDMTKGNLTLLE

RGNANMVKLLLDRGA

IAELGMDKKDVSKKN

AQEEHMLKSESCPFR

RDILNMDKTLKGLNS

GKPGPMGKAGDKGSL

DVQEEMEKTLRQMEE

LHKAIMVKGVDEATI

LILEQMQKDPQALSE

EVTDMMQKALFDFLK

TSFNHMPKIRTLRLH

AYMKNMWKSARKIDA

CKKPSMLKKHIRTHT

GGMTRMPKVQQTVQD

FMASQMLKVSGSATG

LRPWLMEKELMMGVL

VSSDQMAKLRSLFAS

RHGTDMAKYFESLVR

EARKQMSKEEKLKIK

KIWMAMVKVLSGRKP

GQETIMKKVTSVKGP

QALDKMEKEWASILF

....M..k.......

LMAYMASKEEETLDH

PTSYMTGKHLPEQPH

AIWMMDPKDISEFQH

LGLEMTGKLDSNTVE

IISSMAGKMTQPLIA

LPTAMAAKMLLDGEI

AGLLMTLKGLPSTYN

EGHGMDRKTESSLSL

KIMEMAAKGIKPITL

VISSMHAKVDPSLGM

NRVMMVAKTFLDAGH

TDAAMATKTLEKVTS

EMDGMSTKKNVFIIG

VDLDMVLKCLRYYAG

VVMEMASKAIGRLAM

GQGIMVHKHEGMKVF

KATDMTFKAKLYDNH

DQGDMALKMMRLVTQ

EDFEMALKKVSKSVS

GDDPMDFKINTEEIM

ISGKMTLKECGMMDK

GFLDMIDKEQKSTLI

TLTMMQLKQMLPLNT

ASHKMFSKAVSHRLH

NIAHMVAKGAAVSLN

EEECMFPKATDMTFK

GRRGMRPKMMMPFDS

PSPGMSAKNAEKPNM

VQAMMDPKSTAEQRL

FTFEMVIKMIDQGLI

NKCDMDHKRVVPKGK

EMAEMERKNELKLKI

DGNWMTLKDSSRQPP

GSSIMPGKVNPTQCE

EKSSMIRKSLEMLEL

IKTVMFDKTGTITHG

CVAVMEGKQAKVLEN

FSHWMNIKIILDELV

KVGIMYCKAGQSTEE

VGTRMPPKNFSRGSL

KFVPMSAKLDPLIIA

YMRWMNHKKSRVMDF

TMQAMQQKLEDFRDY

KHALMIIKKGNDQPG

LASTMSNKCDVIVVG

FTDTMTFKERVWNHL

ESLAMKEKTISGMKN

PTVVMHLKKQDLKET

LKEPMDQKRYASSPY

MELGMVQKFKIDYET

QVPAMGGKLSEKTAK

HFGSMRGKERELIVL

ACCDMAYKFPFGSSC

IQEEMNEKIERLKAE

LELFMETKQQKRVKE

SGPPMGPKGGAAASS

LANNMKLKQDIARQK

SLDYMVTKIPRWDLD

AAELMQQKEATTEQQ

NPKAMLNKALCNVIA

Please cite:

Chou MF & Schwartz D (2011).

Biological sequence motif discovery using motif-x.

Curr Protoc Bioinformatics. Chapter 13:Unit 13.15-24. doi:10.1002/0471250953.bi1315s35.

Schwartz D & Gygi SP (2005).

An iterative statistical approach to the identification of protein phosphorylation motifs from large-scale data sets.

Nature Biotechnology 23(11):1391-1398.

Parte superior do formulário

Parte inferior do formulário
