# Supplementary material for: Increased chemical acetylation of peptides and proteins in rats after daily ingestion of diacetyl analyzed by Nano-LC-MS/MS
Source: PeerJ. 2018 Apr 25;6:e4688. doi: 10.7717/peerj.4688 (PMC5923218; doi:10.7717/peerj.4688)
Supplement: Supplemental Information 5 — Motif analysis of Lung from control group. Central residue K acetylated. [file peerj-06-4688-s005.docx]

|  |
| --- |

Parameters for this run:

Job Name = **Lung Control Acetylation lysine**

fgtype = 'ms'

fgextenddb = 'ipi.RAT.fasta'

fgcentralres = 'K#'

width = '15'

occurrences = '7'

significance = '0.000001'

bgdb = 'ipi.RAT.fasta'

bgtype = 'fasta'

bgcentralres = 'K'

Job started Wed Feb 3 13:17:32 2016

Results for (**Central Foreground Residue: K# ; Background Residue: K**)

Number of Peptides in Original Dataset: 5114

Number of Peptides in Orignial Dataset that are Unique: 766

Number of Peptides found in Database (ipi.RAT.fasta): 748

Number of Peptides NOT found in Database (ipi.RAT.fasta): 18

Number of central residues (residue = 'K#') mapped to the database : 962

Number of peptides without unique database mappings: 21

Number of peptides too close to protein termini: 26

Final Unique Target Peptides: 849

It took 95 seconds to preprocess foreground dataset

The input file has been converted to a pre-aligned file that may be used for subsequent runs of motif-x.

[Right-click here](http://motif-x.med.harvard.edu/cgi-bin/viewres.pl/motif-x_20160203-30304-29885321_Kx_vs_K-3158_fg.txt?text=n) to save it as a 'pre-aligned' dataset for possibly faster analysis in the future.

It took 6 seconds to preprocess background dataset

**Motifs Found**

| \| **#** \| **Motif Logo** \| \| --- \| --- \| \| 1. \| [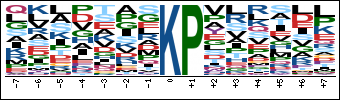](http://motif-x.med.harvard.edu/cgi-bin/jobres.pl?jobid=20160203-30304-29885321#.......kP......) \| \| 2. \| [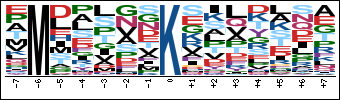](http://motif-x.med.harvard.edu/cgi-bin/jobres.pl?jobid=20160203-30304-29885321#.M.....k.......) \| \| 3. \| [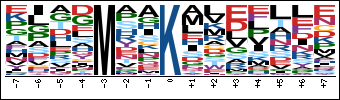](http://motif-x.med.harvard.edu/cgi-bin/jobres.pl?jobid=20160203-30304-29885321#....M..k.......) \| | \| **#** \| **Motif** \| **Motif Score** \| **Foreground Matches** \| **Foreground Size** \| **Background Matches** \| **Background Size** \| **Fold Increase** \| \| --- \| --- \| --- \| --- \| --- \| --- \| --- \| --- \| \| 1. \| **[.......kP......](http://motif-x.med.harvard.edu/cgi-bin/jobres.pl?jobid=20160203-30304-29885321#.......kP......)** \| 9.91 \| 95 \| 849 \| 39069 \| 705467 \| 2.02 \| \| 2. \| **[.M.....k.......](http://motif-x.med.harvard.edu/cgi-bin/jobres.pl?jobid=20160203-30304-29885321#.M.....k.......)** \| 6.40 \| 39 \| 754 \| 13917 \| 666398 \| 2.48 \| \| 3. \| **[....M..k.......](http://motif-x.med.harvard.edu/cgi-bin/jobres.pl?jobid=20160203-30304-29885321#....M..k.......)** \| 6.11 \| 36 \| 715 \| 13076 \| 652481 \| 2.51 \|   Motif search time: 20 seconds |
| --- | --- | --- | --- | --- | --- | --- | --- | --- | --- | --- | --- | --- | --- | --- | --- | --- | --- | --- | --- | --- | --- | --- | --- | --- | --- | --- | --- | --- | --- | --- | --- | --- | --- | --- | --- | --- | --- | --- | --- | --- | --- |

**Raw sequence data** (motifs followed by their matching peptides):

.......kP......

QAIAERIKPVLMMNK

QVAPQRRKPVVSGSL

MKLPKSAKPVSPMRM

QLSDLLIKPVQRIMK

SSVISIMKPVRKRKT

QAAGEVSKPLRAIIC

LSKATARKPHRSEML

QIDVDVSKPDLTAAL

IHHDQPMKPLDRAVF

KVAVKTMKPGSMSVE

SGLDMHTKPWIRARA

LEAPEPRKPVTAQER

GKEGSISKPHLQSRR

MLSIHPLKPEALHLP

RKSLCHAKPIALMAL

WDLILKYKPGRTILL

SIDPKDPKPDSQDLL

QVIDHGRKPTQQEAY

QVPGAVAKPYHRQGA

KKEDLYLKPIQRTIL

AITIDRIKPASVVDR

KLIVALMKPSRLYDA

LSCPVCRKPCSETVL

LFKAIGVKPPRGILL

RTGVEVGKPTHFTVL

GGCGYVLKPPVLWDK

NSAKTARKPEHMAVE

MPSFAPDKPGRIFLM

ITLHSAQKPLRKVCI

KHLPSIIKPYEEQFK

ICKTIGTKPVAHIDQ

GNLDGISKPASSSRS

QRLPVSTKPVEDDVD

AEKDNQMKPKLERII

CKANGRPKPTYRWLK

SVAVKCLKPDVLSQP

RMAASSKKPPISHDD

APSVEKEKPTREDSE

SMKVAIVKPGVPMEI

IDDPTDSKPEDWDKP

RLTLPVIKPPLRHFK

ILLEIGQKPVILTYE

SKKKEKSKPYPGAEL

GGIPVPSKPGTFVKK

KEKDILDKPQSVCCL

IVDMVPGKPMCVESF

LKGAEKGKPSVRQEQ

KLQACCDKPVLQKSQ

APPLTPSKPPVVVDW

EDKDKEGKPLLKAVM

GQTHKPLKPALKRGP

AASFSIFKPSSYHIV

EDSDAEEKPVKQEDF

SVLITAEKPVGDLSS

SKPEDWDKPEHIPDP

VEEVAPAKPPRHLTP

RPSHRARKPPGDRLP

DALRWMPKPWERTGP

GRCNRAGKPVICATQ

IKQLLKDKPEHVGLK

TFTKTIDKPAWLGFL

VLSPTAAKPSPFEGK

WASGVSPKPDPKTIS

GIVEFSGKPAARKAL

PYNPSMSKPDAWGVT

MSAKNAEKPNMQRNN

LKRLEAVKPTVGMKR

ERSIVDYKPNLDLLE

KEEQNRGKPNWEHLN

EPPQQAQKPAVASDY

ALCNGDSKPENAGGD

FTKFHSLKPKLLEAL

GTPHSHTKPYVRSKG

LDMGCSQKPKAEQSE

LLLTDKMKPEEKVAT

LIEHAHTKPLLPSQL

LYARTTSKPRSASGD

PRRRKFHKPITMTIP

RARIDEFKPYIPLIQ

QELENMLKPFGQVIS

SGNAHIGKPAPDFTG

ASDVEDEKPPLPPRS

PSAYGSVKPYTNFDA

IVTLGLAKPSSNISF

KRRVVPQKPPPSPHP

PKPFSAPKPQTSPSP

GAHGQTHKPLKPALK

DMVCSIQKPSRTSPT

QRIHTGEKPFRCSEC

QRIHTGEKPYQCIEC

FVIGKGNKPWISLPR

MYFSDFGKPRFVYLE

AGVGAGGKPGKVPGV

RRIHTGEKPYPCTEC

PQTSPSPKPATKKEP

.M.....k.......

EMDGTENKSKFGANA

TMSSSNGKSVTWAQN

VMDWINDKEAIVTSE

PMPPSASKVAVDSSK

PMGPPGPKGEPGTPG

VMDFLFEKWKLYSDQ

MMPLGVNKSPLPKEP

AMSPDVAKSAAETSA

IMHYGNMKFKQKQRE

HMLFLGTKKYPKENE

SMDELNPKSTVDLLL

AMLSLGSKGDTRKQI

PMILVGNKCDLEDER

EMDGMSTKKNVFIIG

WMIPPEAKESMDKNK

RMMSTGSKKSLLSHE

MMTRSPVKVTLSEGP

VMELLPEKHGQYSLA

AMLSTGFKIPQKGIL

TMDTPAEKLIDYCKR

QMLESMIKKPRPTRA

QMEEINTKEVAQRIT

KMVPTSDKGRFYAFG

KMHAINGKMFGNLQG

TMMDPFRKRLLVPQA

EMMMYHMKVSDDEYT

PMTLLVVKIQPGMAF

YMGASSLKGIQLFDR

PMEIVLNKESRRKTP

TMAADVLKAIQRIMT

AMHASLDKFLASVST

LMDDVDFKAKVTRVE

MMSTGSKKSLLSHEF

DMTIHQGKRWSTASA

LMAYMASKEEETLDH

GMIKAAGKRIYHLNV

NMLQAPVKNVTKYGN

EMQNFVSKQTEMLPQ

EMLREDIKDYYTDLS

....M..k.......

PIALMALKMMELSEE

TIDGMHCKSCVSNIE

HIRHMSNKGMEHLYN

PTTGMDPKARRFLWN

LPAGMSAKMLGGVFK

PSPGMSAKNAEKPNM

ELADMVAKHSDFASK

GVIPMNAKDLEEALE

NIAHMVAKGAAVSLN

KPVLMMNKMDRALLE

NANVMHGKHVMVLEC

FFHTMRYKALDPRNP

TLDYMHLKMCSLHDQ

PPPSMPHKERYFDRI

IATLMPTKDVDKHRC

YDGSMYMKIMLPNAM

MGYMMAKKHLEINPD

VLTDMTEKIVSPLID

KVGIMYCKAGQSTEE

EPLEMAHKVKVVVFD

KCRYMDSKMKPLWLV

KATDMTFKAKLYDNH

YYEAMKQKQTEVLFC

QGLEMRIKLWEELKS

GLHQMGIKYDGNHIP

EEECMFPKATDMTFK

ALSNMPKKSSVPTEE

MHSDMPGKEGSISKP

TIQEMQQKLEDFRDY

EGDDMEHKTLKITDF

VQLQMEAKLWGEEYN

AGGAMSSKFFLMALA

HFGSMRGKERELIVL

FSHWMNIKIILDELV

GSFFMYSKLNVERSR

RDVGMADKSVASGGW

Please cite:

Chou MF & Schwartz D (2011).

Biological sequence motif discovery using motif-x.

Curr Protoc Bioinformatics. Chapter 13:Unit 13.15-24. doi:10.1002/0471250953.bi1315s35.

Schwartz D & Gygi SP (2005).

An iterative statistical approach to the identification of protein phosphorylation motifs from large-scale data sets.

Nature Biotechnology 23(11):1391-1398.

Parte superior do formulário

Parte inferior do formulário
