# Supplementary material for: Increased chemical acetylation of peptides and proteins in rats after daily ingestion of diacetyl analyzed by Nano-LC-MS/MS
Source: PeerJ. 2018 Apr 25;6:e4688. doi: 10.7717/peerj.4688 (PMC5923218; doi:10.7717/peerj.4688)
Supplement: Supplemental Information 6 — Motif analysis of Lung from group treated with 540 mg/Kg/day of Diacetyl. Central residue R acetylated. [file peerj-06-4688-s006.docx]

|  |
| --- |

Parameters for this run:

Job Name = **Lung Treated Acetil R**

fgtype = 'ms'

fgextenddb = 'ipi.RAT.fasta'

fgcentralres = 'R#'

width = '15'

occurrences = '7'

significance = '0.000001'

bgdb = 'ipi.RAT.fasta'

bgtype = 'fasta'

bgcentralres = 'R'

Job started Wed Feb 3 14:23:40 2016

Results for (**Central Foreground Residue: R# ; Background Residue: R**)

Number of Peptides in Original Dataset: 6181

Number of Peptides in Orignial Dataset that are Unique: 1768

Number of Peptides found in Database (ipi.RAT.fasta): 1729

Number of Peptides NOT found in Database (ipi.RAT.fasta): 39

Number of central residues (residue = 'R#') mapped to the database : 2084

Number of peptides without unique database mappings: 33

Number of peptides too close to protein termini: 63

Final Unique Target Peptides: 1694

It took 139 seconds to preprocess foreground dataset

The input file has been converted to a pre-aligned file that may be used for subsequent runs of motif-x.

[Right-click here](http://motif-x.med.harvard.edu/cgi-bin/viewres.pl/motif-x_20160203-17790-66958336_Rx_vs_R-6588_fg.txt?text=n) to save it as a 'pre-aligned' dataset for possibly faster analysis in the future.

It took 6 seconds to preprocess background dataset

**Motifs Found**

| \| **#** \| **Motif Logo** \| \| --- \| --- \| \| 1. \| [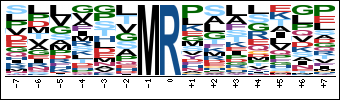](http://motif-x.med.harvard.edu/cgi-bin/jobres.pl?jobid=20160203-17790-66958336#......Mr.......) \| \| 2. \| [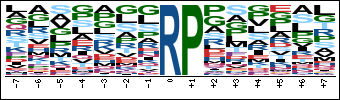](http://motif-x.med.harvard.edu/cgi-bin/jobres.pl?jobid=20160203-17790-66958336#.......rP......) \| \| 3. \| [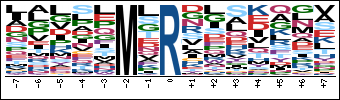](http://motif-x.med.harvard.edu/cgi-bin/jobres.pl?jobid=20160203-17790-66958336#.....M.r.......) \| \| 4. \| [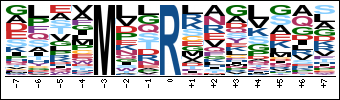](http://motif-x.med.harvard.edu/cgi-bin/jobres.pl?jobid=20160203-17790-66958336#....M..r.......) \| \| 5. \| [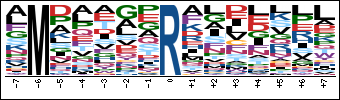](http://motif-x.med.harvard.edu/cgi-bin/jobres.pl?jobid=20160203-17790-66958336#.M.....r.......) \| \| 6. \| [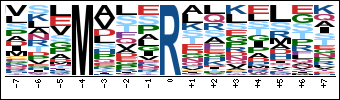](http://motif-x.med.harvard.edu/cgi-bin/jobres.pl?jobid=20160203-17790-66958336#...M...r.......) \| | \| **#** \| **Motif** \| **Motif Score** \| **Foreground Matches** \| **Foreground Size** \| **Background Matches** \| **Background Size** \| **Fold Increase** \| \| --- \| --- \| --- \| --- \| --- \| --- \| --- \| --- \| \| 1. \| **[......Mr.......](http://motif-x.med.harvard.edu/cgi-bin/jobres.pl?jobid=20160203-17790-66958336#......Mr.......)** \| 13.16 \| 76 \| 1694 \| 12186 \| 723483 \| 2.66 \| \| 2. \| **[.......rP......](http://motif-x.med.harvard.edu/cgi-bin/jobres.pl?jobid=20160203-17790-66958336#.......rP......)** \| 10.12 \| 161 \| 1618 \| 41570 \| 711297 \| 1.70 \| \| 3. \| **[.....M.r.......](http://motif-x.med.harvard.edu/cgi-bin/jobres.pl?jobid=20160203-17790-66958336#.....M.r.......)** \| 9.35 \| 66 \| 1457 \| 12890 \| 669727 \| 2.35 \| \| 4. \| **[....M..r.......](http://motif-x.med.harvard.edu/cgi-bin/jobres.pl?jobid=20160203-17790-66958336#....M..r.......)** \| 8.14 \| 58 \| 1391 \| 11759 \| 656837 \| 2.33 \| \| 5. \| **[.M.....r.......](http://motif-x.med.harvard.edu/cgi-bin/jobres.pl?jobid=20160203-17790-66958336#.M.....r.......)** \| 7.82 \| 58 \| 1333 \| 12325 \| 645078 \| 2.28 \| \| 6. \| **[...M...r.......](http://motif-x.med.harvard.edu/cgi-bin/jobres.pl?jobid=20160203-17790-66958336#...M...r.......)** \| 6.31 \| 52 \| 1275 \| 12028 \| 632753 \| 2.15 \|   Motif search time: 24 seconds |
| --- | --- | --- | --- | --- | --- | --- | --- | --- | --- | --- | --- | --- | --- | --- | --- | --- | --- | --- | --- | --- | --- | --- | --- | --- | --- | --- | --- | --- | --- | --- | --- | --- | --- | --- | --- | --- | --- | --- | --- | --- | --- | --- | --- | --- | --- | --- | --- | --- | --- | --- | --- | --- | --- | --- | --- | --- | --- | --- | --- | --- | --- | --- | --- | --- | --- | --- | --- | --- | --- | --- | --- |

**Raw sequence data** (motifs followed by their matching peptides):

......Mr.......

RPPFSTMRPASSQVP

YDVVPSMRPVVLVGP

HKVGSSMRPSLEAQA

PMRGGPMRGGPGPGP

LDLVFLMRPSSMIKV

KMPPASMRPSTSEKE

PSFVSAMRPKTVIGD

PTGAAVMRPMLQSQQ

QPPFLGMRPAAPQYT

DLVVTPMRLAFEKIQ

FPVAGGMRPPQGLIP

VAVIMTMRYSLKVLL

VLLIMVMRLTSMNGE

SLRILQMRPSLSCAS

DGAGEQMRPLLTRGP

MMTHLHMRPSEPKPS

DARGGSMRGSRHHGM

WAFKHTMRNVADTGL

ERLTDLMRMCWQFNP

TMVMTGMRIQIGTQA

EDMYNTMRKMICCAP

VLFFHTMRYKALDPR

KIMTHLMRYFPGADE

PDLVHVMRTLASENI

NVGLHSMRYLHRFGA

LAGRRGMRPKMMMPF

STLKMHMRCHTGEKP

WLLGYTMRFGLYYVD

GCSHTDMRMSDLIPD

SDMMNLMRLEEQLSP

QTDFNLMRVTCRFQE

LGVWLAMRFRNQKDP

IMSDPAMRLILEQMQ

CPPGGGMRKDLCKES

IPHFGSMRGKERELI

PFGRGAMRECFRTKK

SVGVMEMRSPLLAGG

VEHSGTMRSGHYTAY

EKDMIVMRDSFGIRH

PASQTHMRVTASPQS

EMKEDLMRMTAILQT

HNVSPFMRKGTTGDW

VVLDTAMRGGRLGVF

MSSRSVMRAATQTVY

HGVPRVMRFLLLVDV

DEGTPSMRELRSALL

PGIGPLMRDIKNKIC

SVGTFKMRHMPEVPV

SVEWNGMRKMKLGLV

ALDLTGMRPESRLAC

GTVMLDMRASTLDEI

DTAHQAMRVSGRPML

SRGLPGMRGAKGHRG

DEIVTLMREFWVMNT

LSYSSLMRHTGGISP

VNMEVQMRSAPLILA

SLSKPFMRLEKYVTL

GRARPPMRSQASEEE

GPADSVMRQDYRAWK

SILAHYMRRFAKTIG

ERHMSIMRMRIHSNL

KGKRVVMRVDFNVPM

QVSGLEMRKWVDELF

LQQISAMRLHISQLQ

VIPVDIMRRAGIKVT

NDNAEEMRHRFRQLD

RELQEQMRMALEKSR

QPEIKPMRNGCESKE

LQTPVTMRDLFSPVS

RLLADLMRNYDPHLR

THDSEVMREWYQETH

GQAQDSMRKFSDATR

LTLSAPMRLRKTLDL

QNKKKTMRSLSCNSM

ELMLGRMRCLEALGE

IIREENMREGDVTSG

.......rP......

LFMGFMTRPGFAIIT

QAMRVSGRPMLLALL

TTGAVPGRPTMMVST

YFGRKRHRPIMIGIG

LYPGALGRPPPMPPM

LAAGPALRPDMSYAE

IVMAATNRPNSIDPA

RRGRGGGRPPPVCSG

VLMSHLGRPDGVPMP

DRVSVTIRPGMTLLM

QMSLLLRRPPGREAY

SNAPRLIRPYMEPIL

AYAAFNNRPPSTWLT

YTLAAMHRPGTGKLT

LVVGPSSRPPPGLSL

SSAAAGSRPGDGGSL

FYSEILGRPTTVDET

HGGPHGHRPHDVPSH

AMPSGVYRPPGARLT

LAAGILLRPESVRAA

RSMESNGRPASTHYY

WAVHNGTRPPLIKNL

FMYEFEYRPSFVSAM

MVGGGGHRPHEGPGG

FIIGATNRPDIIDPA

YHQLMGHRPQLENLL

VASSVGGRPSRSGLL

QNMPGAIRPAAPRPP

KVLMDGVRPSSPEAL

MVRGGMYRPYGSGGV

GMDRQNRRPGAGGKT

TEVRAMQRPPPGVKL

VVEGFYGRPWVMEQR

EAKSLGIRPNYIDIY

KHSSPVGRPSIGSAS

TATSPTKRPPTKPSV

SGSGVLGRPVMEKEE

LCAQGLVRPALGNAG

ASKEAGPRPPGSGPG

TKLRLSNRPAFMPSE

DFVLEFPRPMMPNMV

KVKMEYFRPNIVVSG

QAAESQLRPWLMEKE

TEVIKAERPDGLILG

SFVKSHIRPYMDEIV

WAAQGQPRPPCSCAV

QHTNSLSRPNSFERS

VKKPHRYRPGTVALR

RVALHQGRPREIINT

LYVQALFRPVAMMVP

IAELSGNRPLTVIMH

RHPEMLLRPENCPAQ

KLSRLWSRPAALMST

SMAHGRARPPMRSQA

AIRPAAPRPPFSTMR

GSEAHDKRPPMLTSS

ALRVKPIRPIGMFSG

FLFPASSRPSGSETA

QSMLGNLRPAHLGPC

DNPSGSTRPASLMFN

GHSNGNRRPESDEDS

HVMGPPLRPDPSTPG

KEKEKKKRPMSQISG

RCTWQLGRPMADSPH

QASDRVLRPMCFVKQ

LSDSPDGRPGGFWRR

QGPAPLRRPDSSDDR

GNPGRDGRPGYQGEQ

QHLVTDSRPGLIEQR

KPSTAEPRPPAGEAQ

RWTAQGARPHPFQNM

HGGDMSNRPVCRHFM

QLLPPTDRPMSPRSL

NLRIASGRPYNPSMS

DPKPHSNRPGAVVRA

DMGRHETRPHPQASP

LANSSALRPGDALNL

GTSIQSLRPKSYVKT

LRQIQVSRPWALAVR

AHPSLYRRPRPIEGL

GSQGESGRPGPPGPS

MSTEASARPLRVGSR

VGMEDGERPRRRRHG

FGKIVAERPGTNSTG

GESGRPGRPGERGLP

NFRTKAARPGKVNGM

PERQRSLRPHHDAST

KNIASMVRPGGLLVI

FMPDSFLRPGGDHDC

QALIRVLRPRNKRRH

ARSIAVTRPNNLVHF

FDLPKRKRPASPALL

YQVYVRLRPFFREFL

PLGLLGVRPGMPPQP

GGVVFHYRPGSTRYS

ARLVEAGRPFIKEAS

STPDDLLRPSTELAT

GAVVRAHRPSAQSLH

VWPLNLLRPRGGPGY

QNGGMCLRPQFCVCK

YFREKKSRPSSTSSG

TAQRLLQRPRPGKAK

IPVTGRRRPYGRDAL

KRVQDLSRPKKQWGT

SGTKKSRRPPGKTGL

SVLVMTVRPEDHGAR

APPAPGPRPPPRAAG

RQAGFPRRPPPRGTR

IGIQQSFRPRFLGVA

GQGASDLRPALAGAS

FDCLLSGRPTAPRAS

PASNQPKRPPTAPEN

SGPGPSLRPQNSTTW

AMVFPYHRPSPGSES

TTATNMDRPRTPGHQ

PEGHELCRPEAVEAE

NRAVANSRPAKAAVH

YKQNGRIRPIVAENP

ALRQGADRPLGGFHG

IARIDTPRPLVGRLI

FSRLLSARPGLKLLA

IRTLSGSRPPLLLRL

LRAWLAGRPEREFYR

DARAPEKRPHTAPFD

ELASAKVRPQANISL

KILSRKRRPNLGRLT

EISYTGSRPASNMVI

TAPCLSSRPALPRQR

EDIVGTARPDEKAIM

STQTMGPRPAAAATA

PPSMACTRPPSAPRN

RNYDPHLRPAERDSD

EGEYDAERPPSKPPP

PEGHPTPRPASPDPG

GLSPLAPRPWRWLLL

IQSLYGARPSSDATV

MKIRERNRPEVFEVI

PNLPPFQRPEAEAMC

VWQEGPARPSARQAV

SCGFVTARPLSIKIV

RKFGVISRPSISKTP

TGGFIRSRPGVPHPD

LSSEEVARPRRSTPT

PWALAVRRPLQEHVR

SSFRIRHRPHDQLRL

GHLPPGPRPLPLLGN

DLSMNKQRPRDSCCP

RQEINYTRPVIILGP

TTTPTDPRPLTSPLR

KKSCLQCRPPNPPES

LRHAMEQRPSQELLW

LGDFKPYRPGQAYVP

ARVPRPERPDLVFEE

RLLSRPQRPQRLWSL

EDIKPLKRPRDEDDA

RRNLDIERPTYTNLN

LKFQREERPLFPQIL

AGTPSFSRPIPTTQK

CCLTKSSRPRVLLRW

TECTTIERPFCMYDA

PSFSSPQRPKHVVGD

.....M.r.......

IGDFGMTRDIYETDY

PIESLMTRCWSKDPS

MEMCSMKRGLDVQME

QLIPHMLRVFMHDNS

AMFPTMNRRGAIKQA

IQLVGMLRGIASGMK

GNPGPMGRKGHKGHP

ILVGGMTRMPKVQQT

SKLEGMFRDMSISNT

NPLPSMTRHFLLMQE

NAQDNMGRCPLHAAV

NAEFQMLRRTSINVV

GVNASMPRVSYIRAV

PGISTMSRGDLSQRA

TAKSDMNRHLHEYME

TTDNIMLRVFPDGHV

DILMYMDRVYVQQNN

YTLWWMLRRSLKKYS

RGLIDMVRNQAMADA

DACNLMARARHQQSH

CLNCQMQRALGMDMT

LGMSFMNRSSGLRNS

QQTTEMLRVMLQQGE

DGGLLMGRDRDKPFK

SHPSMMYRSQSELQS

MDGDEMTRIIWQFIK

GPLSPMSRAGNMGVG

SVLRPMVRGGMYRPY

ENDCVMSRIGATRGL

DLAPQMLRELQETNA

NDDTEMLRSHSEYLN

ATVVMMTRLEEKSRV

QMVEFMQRCASHMKA

IPVDIMRRAGIKVTV

TEISEMNRNISRLQA

LAAIIMKRDPEDMGV

GYFHFMGRTDDIINS

LPHFLMGRYEYETTI

QAYNPMARDLFNAAF

FIYLIMLRRFKQKAH

LSPKSMSRNSSIASD

FLNVPMFRNVSLKCL

GPGSPMSRSQEYLLD

SSQNVMIRHGQTQEY

HLSGGMQRKLSVAIA

IGDVEMKRVLSCPRC

SHHAQMVRSDQTSSP

LVLLLMPRLICKAEL

VAKKQMDRLWHTSSV

WQKILMVRSLVVSPH

PLHAMMERGPQTLKE

RSVQLMERRMDKAGQ

AFIDFMSRETADTDT

KRYQTMERIWRKIMK

AEKPNMQRNNTLGIS

IPPQRMLRRQAAFEL

ERHRQMIRNYQYQLL

VVGLTMPRYCLFGDT

PVSIWMFRTDFVLEF

FTSLLMERLSVDYGK

NVPYVMSRGATPYGG

AIIRAMGRLKIDFGE

DMGTAMARDERVNLL

LRVERMDRIEKERKH

EEMAEMERKNELKLK

LKGGHMDRQRRDFLR

....M..r.......

GLQCMLNRLAGVKDF

ETVEMTLRRNGLGQL

ESVEMTLRRNGLGQL

GPAWMVRRSEGHEQT

DPELMLGRMRCLEAL

GGSVMIQRVNGSLAV

AIDFMVDRLESLGQS

DPRAMVGRLNDLLVK

WLTAMWSRSFSLAAN

DFEMMLQRKKSMCGK

SLKPMKTRVVGGIQK

ALGIMNNRLAETSAS

GLAVMPLRDVAAKGS

NISSMLGRMANPARS

DLEQMPQRRRMSSTG

GFSYMWERAQGSVAL

MFPTMNRRGAIKQAK

AITGMPTRKANNGFP

PSDMMDGRVEAIKAA

EYTCMIPRDTHDGAF

SCVMMQGRFHMYEGY

KYPIMKQRGFYSDIL

ILGPMKDRINDDLIS

DDVNMPAREVYGAQP

DGTAMVTRAGLPCQD

KTIFMWYRSKERGQR

AQPQMQGRQAVPGPQ

ILHGMGLRLLALGHH

QQAVMSSRCFQLSEG

LQSPMISRVNAGKGD

STHTMPVRRNRKSSS

EARKMVSRCTYLNII

QHRHMLVRMAFQDGT

IYLIMLRRFKQKAHV

VPSVMVSRSHRDAGA

TQEFMDLRTRYTALV

CQKAMQDREVSKSDI

HKPVMALRREDVNAW

KAIEMELRQMEVAQA

APGPMGPRGAPGERG

SVGFMDTRKRHQSDG

ECTDMGLRCGKVRDR

SSLDMNGRCICPSLS

GLRWMKARADVGSIG

TLWWMLRRSLKKYSF

VHVGMDDRRRDSPRA

PSNLMLDRLSGKILH

SFLCMENRTQTLKPT

KHEGMSERERQVMKR

GAALMRNRDLQLEVD

PSAMMSSRMGPSQNA

NAKRMETRGAGVTLN

FTEIMNQREQFYHKV

HFLLMQERIVKVMSD

ENQAMDTRLQLAMVC

RPWVMEQRKELFRRL

GINSMLQRKIAELEE

AEFQMLRRTSINVVR

.M.....r.......

GMDPSARRFLWNNLL

CMPTEGQRVINEQSL

FMPFSAGRRACLGEP

FMPFSAGRRLCLGEP

GMQLFGGRFNFNDGT

SMDQYGGRHGGGSGG

SMAYAGARFVFSLVD

NMVNTGHRKGVTVPK

LMQAEVNRFQDTKRL

SMDATSERDFVAEFL

DMPGYGYRAPEDFVD

AMAAINARKMKFALL

FMLQVSPRLTFLSLL

RMIVTGHRRIHISRQ

AMKGAGTRHKTLIRI

AMVHEVQRYIDIGPN

GMAAPQSRAPEGKPI

KMSTSGPRAFSSRSF

VMDNHRERIANFKIE

QMAAQQKRELMNHHL

GMKLAQTRAILNYIA

GMSQQDSRNMNYVML

AMKPIDGRKLLDSLM

PMHLTGSRSLIHSRA

RMIEDAERAGTLKPG

LMDGLKQRAHVIVMA

LMDLECSRDGLMCEQ

KMAMDAGRRDNLRSY

WMAAEVIRMQDPNPY

AMITAALRMFMELGM

VMDKNNDRETDFVLW

PMNLVLFRFAIEHIS

QMFTHYDRAHIAQLC

SMNKQRPRDSCCPVL

EMDELCYRDGEEYEW

LMNAAGSRLLFVLHG

FMGSHFDRHYCGKCC

VMEFVLPRFGVDVKV

AMLHTLERINSDPTL

RMDFFYNRSNTDTAD

NMGQWKIRAFYEHAP

AMPVFHTRTIESILE

TMPVGDFRKAIIQQT

HMPEVPVRIRIGLHT

AMVDIVKRYNWTYVS

YMLQDTLRFLVQDSL

LMSSGLPRETLGQIW

KMTIEPPRGVKANLL

PMHEYAERLSALLPE

AMTLCYARVSRELWF

HMSVWEQRTSQLRRH

LMFNTEERKCSQTHK

IMLSASPRMSGFMYQ

SMWPIEARNAAIANH

NMGMDFCRQDEKCDY

KMKPPSQRCVSPREK

FMEDGTARQRLRGPA

QMRPLLTRGPDEEAV

...M...r.......

VRLMLLERLLQTLPQ

VPLMAFPRIESPLET

VALMVQERFGKSLLE

VVEMLLDRAAPILSK

CSVMYFSRGFQMLGP

PGPMAPPRAPGPLSK

VSFMVDARGGSMRGS

SPEMHSPRLSGERSR

LPPMVGSRKSKGSGN

QTSMSTPRTLRKLGR

VLEMGAARHFLRMQQ

RKEMSGSRSSPKLEY

MTVMSLSRDLKDDFH

SLSMAHGRARPPMRS

ISSMEGERLREEMEE

PRVMSTQRVANTSTQ

CNLMARARHQQSHFD

LRRMVQEREKDILAA

DKEMAEERAESLQQE

AWGMVSNRDTLIRNC

KKLMLAVRKLAELQK

SLAMDFSRQSPDHPT

VSSMALARSQCRNSP

ASPMTVPRKHWWTDA

YKKMASEREGSGSSS

PLVMHQLRCNGVLEG

ISIMKPVRKRKTATI

STLMALIRTALDIKI

IAGMVVTRLDEIEGN

GEVMAIGRTFEESFQ

QLWMVLQRSLVTVRR

NYGMPFSRTEDGRIY

PIQMQTPRARRAHRP

RHVMDSVRQAKLAVQ

VALMKPSRLYDAYEL

MVEMLTDRNLNLEEK

LKNMGSIRYQHLMTI

FDLMYAKRAFVHWYV

DIAMHLNREILQLPL

LDPMTSYRSKGWQVK

SRMMPSTRDSTSTPA

FDMMLTGRNIRADRA

NTTMDEFRQHLQATG

GEDMDDERLKELFGK

THTMPVRRNRKSSSS

MNNMQQLRVQLEKMF

KNEMQVLRKTRHVNI

LNAMVYIRGHAEDYN

EADMIKLRTQVTITT

HVGMDDRRRDSPRAT

DFYMRRFRSQNGSLG

AGSMEEKREVFSYLV

Please cite:

Chou MF & Schwartz D (2011).

Biological sequence motif discovery using motif-x.

Curr Protoc Bioinformatics. Chapter 13:Unit 13.15-24. doi:10.1002/0471250953.bi1315s35.

Schwartz D & Gygi SP (2005).

An iterative statistical approach to the identification of protein phosphorylation motifs from large-scale data sets.

Nature Biotechnology 23(11):1391-1398.

Parte superior do formulário

Parte inferior do formulário
