# Supplementary material for: Increased chemical acetylation of peptides and proteins in rats after daily ingestion of diacetyl analyzed by Nano-LC-MS/MS
Source: PeerJ. 2018 Apr 25;6:e4688. doi: 10.7717/peerj.4688 (PMC5923218; doi:10.7717/peerj.4688)
Supplement: Supplemental Information 7 — Motif analysis of Lung from control group. Central residue R acetylated. [file peerj-06-4688-s007.docx]

|  |
| --- |

Parameters for this run:

Job Name = **Lung Control AcetylatiR**

fgtype = 'ms'

fgextenddb = 'ipi.RAT.fasta'

fgcentralres = 'R#'

width = '15'

occurrences = '7'

significance = '0.000001'

bgdb = 'ipi.RAT.fasta'

bgtype = 'fasta'

bgcentralres = 'R'

Job started Wed Feb 3 14:02:20 2016

Results for (**Central Foreground Residue: R# ; Background Residue: R**)

Number of Peptides in Original Dataset: 5114

Number of Peptides in Orignial Dataset that are Unique: 807

Number of Peptides found in Database (ipi.RAT.fasta): 788

Number of Peptides NOT found in Database (ipi.RAT.fasta): 19

Number of central residues (residue = 'R#') mapped to the database : 1055

Number of peptides without unique database mappings: 11

Number of peptides too close to protein termini: 44

Final Unique Target Peptides: 876

It took 46 seconds to preprocess foreground dataset

The input file has been converted to a pre-aligned file that may be used for subsequent runs of motif-x.

[Right-click here](http://motif-x.med.harvard.edu/cgi-bin/viewres.pl/motif-x_20160203-13262-03543199_Rx_vs_R-2359_fg.txt?text=n) to save it as a 'pre-aligned' dataset for possibly faster analysis in the future.

It took 5 seconds to preprocess background dataset

**Motifs Found**

| \| **#** \| **Motif Logo** \| \| --- \| --- \| \| 1. \| [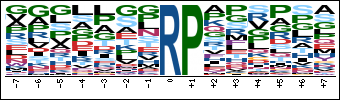](http://motif-x.med.harvard.edu/cgi-bin/jobres.pl?jobid=20160203-13262-03543199#.......rP......) \| \| 2. \| [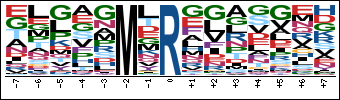](http://motif-x.med.harvard.edu/cgi-bin/jobres.pl?jobid=20160203-13262-03543199#.....M.r.......) \| | \| **#** \| **Motif** \| **Motif Score** \| **Foreground Matches** \| **Foreground Size** \| **Background Matches** \| **Background Size** \| **Fold Increase** \| \| --- \| --- \| --- \| --- \| --- \| --- \| --- \| --- \| \| 1. \| **[.......rP......](http://motif-x.med.harvard.edu/cgi-bin/jobres.pl?jobid=20160203-13262-03543199#.......rP......)** \| 14.61 \| 114 \| 876 \| 42253 \| 723483 \| 2.23 \| \| 2. \| **[.....M.r.......](http://motif-x.med.harvard.edu/cgi-bin/jobres.pl?jobid=20160203-13262-03543199#.....M.r.......)** \| 6.70 \| 38 \| 762 \| 13142 \| 681230 \| 2.59 \|   Motif search time: 17 seconds |
| --- | --- | --- | --- | --- | --- | --- | --- | --- | --- | --- | --- | --- | --- | --- | --- | --- | --- | --- | --- | --- | --- | --- | --- | --- | --- | --- | --- | --- | --- | --- | --- |

**Raw sequence data** (motifs followed by their matching peptides):

.......rP......

HGGDMSNRPVCRHFM

GVGDSGLRPPMVPRQ

GGGSGGVRPEGSPKA

HGGPHGHRPHDVPSH

MVGGGGHRPHEGPGG

AAGFLSPRPFPRAAA

TKLRLSNRPAFMPSE

VGMEDGERPRRRRHG

FGTAAPLRPGMPPTM

RRGRGGGRPPPVCSG

EDHDFTMRPAFGGPA

IVMAATNRPNSIDPA

GGSGTSSRPSSNRSY

NGQLLFDRPMHVKMD

AGSGRTMRPPSPPHV

GPPLTSFRPQCSFMG

GGVTPEGRPPLLDTQ

VSSLISGRPCPGGPA

MASSPSGRPQLSNCS

AAKALGPRPRGRPSV

GALACALRPAGSQAA

GDFFPPERPQQLPHG

PRTLNFNRPFMVVIT

APPAPGPRPPPRAAG

NELEPLNRPQLKMPM

QADHLEVRPAPVSHA

VIVLTGWRPGSGFTN

TAPGGHPRPKPGPPQ

GLLLLGSRPAGGTGP

WAHKPEDRPTFVALR

PGGDAVDRPFQCACC

GNLSDESRPKWKHSN

VPGAAAFRPMGPAGP

RAHSASKRPITCPEC

SGQWDMMRPQKGSIS

DMVLDKLRPENVRVA

RPKDRKHRPHHHHHH

GPAAQYQRPGMSPGS

DYWDPSQRPKLSPSK

EAKSLGIRPNYIDIY

PGDEPDDRPERRPRP

EVLTPDGRPIQMRIG

HKVFEGNRPTNSIVF

FIIGATNRPDIIDPA

DNPSGSTRPASLMFN

DDQKQSGRPYSNNPF

MATPLLMRPLSMDNM

KISANSTRPARWHSK

LDALKNNRPSEGPLQ

KMYGLGTRPYFHSSF

NLRIASGRPYNPSMS

LNGSLQERPMGSRLQ

WAEILVRRPTGNTSW

PPRIPAGRPVMIVVE

EKPPPTGRPPAPPRA

LLGLGTSRPNPRCAE

VFPSIVGRPRHQGVM

PFAALSVRPSTQPRP

LTGVFAPRPSTGPHK

GPNLSTTRPIQQDLG

WPRLGSSRPPIRLPY

SSPDHLNRPINGNGK

DFQLAESRPKELYTE

VSGRHKFRPSFPASS

GPEPGPLRPAHRAGS

WALRFSVRPLSCSSQ

ARTLGLRRPVRGTSM

VFPCVPERPGMPCPG

DNSIHWERPQKPTKS

LHSILGARPPPVKRL

NTFRCTYRPVMEGPH

NRLYMKARPFPDGLA

AGPPAQQRPPPQGGP

KTLTTETRPQPHFNR

RQLSIDTRPFRPASE

WECKANGRPKPTYRW

REAVPHSRPYMVSLQ

MVVNDAGRPKVQVEY

VGANPKVRPNPARFL

PDGKTMLRPVLRLTS

QGRMKFTRPLFKDLA

SDLLQPERPPEARHA

PLHPPARRPHGHRRN

RGREPEARPKRELRE

LAQQMENRPSVQAAL

VIVDDRGRPSGKGIV

ETIEKGKRPAVSMSP

AKHGSSGRPRDPNTC

ANLKLGERPPALLGS

SKKAIKLRPIAVIKG

YRLTLVKRPAEPGTS

ETSQVILRPGPSPNP

QESLTLHRPGGRTAH

VRPSTQPRPDPDSWG

CRVEGMPRPTVSWIL

PSFVSAMRPKTVIGD

RPSVTAPRPGGDAVD

RGRGALTRPVLTKEQ

SRTSSYVRPQTLQAA

ALAIREPRPTSCQTL

GLSPLAPRPWRWLLL

TTTPTDPRPLTSPLR

RLGVVRRRPKDDGPY

ESMIKKPRPTRAEGS

SSVEEGLRPELSQLE

PVSQQWARPRFTQPS

KHQFILSRPQDLRVC

IGIGEPSRPSEKRRT

SVEHRTRRPHSREKH

SIDTRPFRPASEGNP

SLEQVKRRPAHLMAL

IIDPAILRPGRLDQL

GVACEPHRPRQNSEV

LRVLRVLRPLKTIKR

.....M.r.......

GPGHMMNRGGMSGRG

ELGANMYRGGDGPLH

IQLVGMLRGIASGMK

ISGAGMERMGAGLGH

GLGHGMDRVGSEIER

RMGLSMDRMVPTGMG

GPLSPMSRAGNMGVG

GGMENMGRFGSGMNM

GGGTGMDREPVGLEE

DTGVGMTREELVKNL

EEHRGMLREDAVLEY

VKVPMMNRLGMFDMH

VVGVRMPRYCLFGNN

TTDNIMLRVFPDGHV

RLTSAMTREVATDHK

EEPGPMVRGVGGTPR

AEKPNMQRNNTLGIS

LADFGMTRRVGCRVK

QPPASMGRGRDWNVD

EMQGYMMRDLNSSID

AASGGMTRRNTYVCS

GNREWMIRNGLVISN

SLLGRMLRFEPSARI

AGLQSMMRQFQQGAA

TMPAAMYRLLTAQEQ

TQEAFMKRAVANCQA

ENVFAMLRGYRIESY

QLKMPMERALGEVYV

EIYTDMGRFTIAAKH

NYLYRMLRSLLSAQG

MDPSDMMREIRKVLD

NLQLLMERFFRNECR

ACYENMKRTLNDVFS

LSAVMMLRHMGLFDH

TRTPLMVRKIAVRRV

DLMFHMQRQRKLPEE

NTKSEMARHLREYQD

TFLEHMCRLDIDSAP

Please cite:

Chou MF & Schwartz D (2011).

Biological sequence motif discovery using motif-x.

Curr Protoc Bioinformatics. Chapter 13:Unit 13.15-24. doi:10.1002/0471250953.bi1315s35.

Schwartz D & Gygi SP (2005).

An iterative statistical approach to the identification of protein phosphorylation motifs from large-scale data sets.

Nature Biotechnology 23(11):1391-1398.

Parte superior do formulário

Parte inferior do formulário
