# Supplementary material for: Increased chemical acetylation of peptides and proteins in rats after daily ingestion of diacetyl analyzed by Nano-LC-MS/MS
Source: PeerJ. 2018 Apr 25;6:e4688. doi: 10.7717/peerj.4688 (PMC5923218; doi:10.7717/peerj.4688)
Supplement: Supplemental Information 8 — Western Blotting individual data. [file peerj-06-4688-s008.pdf]

SM 8- Western Blotting individual data

**Replicate**	**Control Group**	**Group treated with 540 mg/Kg/day of 2,3-butanedione**
1	0,100206272	0,448102679
2	0,103843601	0,337189788
3	0,104928188	0,268296379
**Mean**	0.1030 ± 0.001428 N=3	0.3512 ± 0.05238 N=3
**Difference between means**	-0.2482 ± 0.05240	
**95% confidence interval**	-0.3937 to -0.1028	
**R squared**	0,8487	
**P value**	0,0015	
